# Sleep spindling and fluid intelligence across adolescent development: sex matters

**DOI:** 10.3389/fnhum.2014.00952

**Published:** 2014-11-28

**Authors:** Róbert Bódizs, Ferenc Gombos, Péter P. Ujma, Ilona Kovács

**Affiliations:** ^1^Institute of Behavioural Sciences, Semmelweis UniversityBudapest, Hungary; ^2^Department of General Psychology, Pázmány Péter Catholic UniversityBudapest, Hungary

**Keywords:** sleep spindling, EEG, adolescence, gender, IQ, Raven Progressive Matrices Test, sigma waves

## Abstract

Evidence supports the intricate relationship between sleep electroencephalogram (EEG) spindling and cognitive abilities in children and adults. Although sleep EEG changes during adolescence index fundamental brain reorganization, a detailed analysis of sleep spindling and the spindle-intelligence relationship was not yet provided for adolescents. Therefore, adolescent development of sleep spindle oscillations were studied in a home polysomnographic study focusing on the effects of chronological age and developmentally acquired overall mental efficiency (fluid IQ) with sex as a potential modulating factor. Subjects were 24 healthy adolescents (12 males) with an age range of 15–22 years (mean: 18 years) and fluid IQ of 91–126 (mean: 104.12, Raven Progressive Matrices Test). Slow spindles (SSs) and fast spindles (FSs) were analyzed in 21 EEG derivations by using the individual adjustment method (IAM). A significant age-dependent increase in average FS density (*r* = 0.57; *p* = 0.005) was found. Moreover, fluid IQ correlated with FS density (*r* = 0.43; *p* = 0.04) and amplitude (*r* = 0.41; *p* = 0.049). The latter effects were entirely driven by particularly reliable FS-IQ correlations in females [*r* = 0.80 (*p* = 0.002) and *r* = 0.67 (*p* = 0.012), for density and amplitude, respectively]. Region-specific analyses revealed that these correlations peak in the fronto-central regions. The control of the age-dependence of FS measures and IQ scores did not considerably reduce the spindle-IQ correlations with respect to FS density. The only positive spindle-index of fluid IQ in males turned out to be the frequency of FSs (*r* = 0.60, *p* = 0.04). Increases in FS density during adolescence may index reshaped structural connectivity related to white matter maturation in the late developing human brain. The continued development over this age range of cognitive functions is indexed by specific measures of sleep spindling unraveling gender differences in adolescent brain maturation and perhaps cognitive strategy.

## Introduction

Adolescence is a critical period in the maturation of the neural architecture and in the related development of cognitive functions. This period is characterized by the late maturation of association areas involved in top-down control of thoughts and action (Casey et al., 2005). New findings in developmental psychology and neuroscience reveal that a fundamental reorganization of the brain takes place in adolescence (Konrad et al., 2013). The major reorganization of cortical networks during adolescence is indexed by the changing patterns of synchronous, oscillatory activity (Uhlhaas et al., 2009). Moreover, evidence suggests profound changes in the organization and function of cortical networks during transition from adolescence to adulthood (Uhlhaas and Singer, 2011). These changes may have substantial implications for the understanding of cognitive functions and cognitive development (Uhlhaas et al., 2009; Uhlhaas and Singer, 2011).

Intellectual ability is closely related to cortical development in children and adolescents. The level of intelligence is associated with the trajectory of cortical development, primarily in frontal regions implicated in the maturation of intelligent activity: vigorous cortical thinning by early adolescence is a positive index of IQ (Shaw et al., 2006; Gogtay and Thompson, 2010). Furthermore, results emphasize the possibility that an individual’s intellectual capacity relative to their peers can decrease or increase in the teenage years. Decreases and increases were found to depend on structural and functional changes of specific brain regions (Ramsden et al., 2011).

Striking sex differences in the functional architecture (Ingalhalikar et al., 2014) and developmental trajectory (Simmonds et al., 2014) of the brain of children, adolescents and young adults were established recently. The above cited studies suggest that males and females are characterized by modularity and cross-modularity of the neural architecture as well as linear and non-linear white matter growth, respectively. In addition, gender roles were also shown to have a modulatory effect on regional brain volumes of children and adolescents (Belfi et al., 2014). Striking sex differences in the neural correlates of intelligence were reported in terms of waking electroencephalogram (EEG; Neubauer et al., 2002; Jausovec and Jausovec, 2005) and brain anatomy (Gur et al., 1999; Haier et al., 2005): neural connectivity measures and white matter structures are reliable neurobiological correlates of intelligence in women but not in men. Consequently, sex and gender are of primary interest when investigating the brain-derived factors related with adolescent neurocognitive development. Neural connectivity and white matter-related indices expressing cross-modular brain organization are candidate neurobiological markers of cognitive efficiency in females, but not in males.

Sleep EEG is considered to be the clearest window through which to view adolescent brain development (Colrain and Baker, 2011a). The sleep EEG changes during adolescence were considered as indexes of fundamental brain reorganization (Feinberg and Campbell, 2010). As Colrain and Baker (2011a) acknowledged, EEG power reflects the sum of inhibitory and excitatory postsynaptic potentials in thousands of neural columns sampled by an individual electrode, and the curve describing changes in delta EEG over the lifespan is remarkably similar to those based on postmortem anatomic synaptic density measures and cerebral metabolic rate (Feinberg and Campbell, 2010). Most of the known sleep architectural or quantitative EEG measures strongly and reliably depend on the chronological age of the adolescent subjects. Reports on developmental changes in human sleep most frequently emphasize age-related increases in Stage 2 (S2) sleep percentage, and decreases in slow wave sleep (SWS) percentage. The above changes are reflected in age-related decreases in quantitative EEG measures of sleep EEG delta and theta waves during both NREM and REM sleep (Ringli and Huber, 2011; Colrain and Baker, 2011b; Feinberg and Campbell, 2013).

Sleep spindles are groups of rhythmic neuronal oscillations in the frequency range of sigma waves (11–16 Hz), constituting the hallmarks and major defining features of NREM sleep (De Gennaro and Ferrara, 2003; Lüthi, 2014). Hypotheses on the preferential involvement of sleep spindles in sleep-related neural plasticity (Timofeev et al., 2002), offline information processing (Fogel and Smith, 2011), and sleep protection (Dang-Vu et al., 2010) have been put forward. Individual profiles in sleep EEG spindling reflect the microstructural properties of white matter tracts as measured by diffusion weighted magnetic resonance imaging, with high levels of spindling being related to high axial diffusivity in white matter structures (Piantoni et al., 2013). Moreover, sleep spindles were shown to constitute a physiological index of overall mental efficiency or intelligence (Fogel and Smith, 2011). Several studies emphasized the differences between the frontally and centro-parietally dominant slow (~11–13 Hz) and fast (~13–16 Hz) spindles (SSs and FSs), respectively (De Gennaro and Ferrara, 2003). Apart from frequency and topography other differences in specific features characterizing spindle types are seen in the hemodynamic activities indexing neural activation patterns associated with SSs and FSs (Schabus et al., 2007). Moreover, increasing evidence supports the thesis on the specificity of the cognitive correlates of SSs and FSs: SSs were shown to correlate with visual perceptual learning (Bang et al., 2014), while FSs with more complex abilities and processes, like fluid intelligence (Bódizs et al., 2005), visuospatial memory (Bódizs et al., 2008), learning ability (Lustenberger et al., 2012) and word-location associations (Cox et al., 2014).

In spite of the hints on the potential significance of sleep EEG spindle measures in unraveling the details of the neurodevelopmental processes of adolescence (Tarokh et al., 2011), there are only a few controversial reports focusing specifically on this issue. Although it was claimed that sleep spindle activity changes with maturation until the age of 16 years in terms of length and density (Scholle et al., 2007) there is only scarce data on late adolescence or on the transition from adolescence to adulthood. In contrast to Scholle et al. (2007), Shinomiya et al. (1999) reported a decrease in the power of slow sleep spindling until the age of 13 years, but little change in the power of fast centroparietal spindles between 4 and 24 years. These controversies might result from an inappropriate methodological approach of the individual-specific and developmentally changing frequencies of SSs and FSs. Given the finding on the relationship between individual level of sleep spindling and white matter integrity (Piantoni et al., 2013) as well as the continuing white matter development during late adolescence (Peters et al., 2012) the issue of adolescent development in sleep spindling is of utmost importance. The potential significance of a detailed analysis of sleep spindling during adolescence is further supported by the correlations of specific sleep spindle measures with late developing, higher order intellectual performances of preadolescent (Geiger et al., 2011, 2012; Chatburn et al., 2013; Gruber et al., 2013) and adult human volunteers (Bódizs et al., 2005, 2008; Schabus et al., 2006, 2008; Lustenberger et al., 2012). According to our knowledge, no data on the sleep spindle-intellectual ability relationship in adolescents was published in the literature. Thus, the potential relevance of the above mentioned sleep spindle-related EEG indexes in revealing the individual patterns of cognitive development remained largely neglected in previous reports.

In summary adolescence is a critical period of brain maturation and cognitive development, presumably characterized by increasing sexual dimorphism and gender-divergence. In spite of the fact that sleep EEG was acknowledged as a prominent route in discerning the neurodevelopmental processes of adolescence and individual-specific measures of sleep spindling were shown to reflect complex cognitive processes and faculties in both children and adults, no prior study explicitly addressed the neurocognitive developmental aspects of sleep spindle oscillation in adolescents. The corroboration of the above cited evidence for a positive association of fast sleep spindling with complex, human-specific cognitive performances and faculties in both children and adults, with the unequivocal growth of white matter structures in the adolescent brain, and with the relationship between white matter integrity and sleep spindling lead us to hypothesize that fast sleep spindling correlates positively with chronological age (H1). By completing the above considerations with the claim suggesting that white matter is the major determining neural substrate of thinking in women, but not in men we further hypothesize that fast sleep spindling predicts overall mental efficiency as measured by intelligence tests primarily in females (H2).

Hypotheses were tested in a home polysomnographic study focusing on the effects of chronological age and developmentally acquired overall mental efficiency (fluid IQ) with sex as a potential modulating factor.

## Materials and methods

### Subjects

Subjects (*N* = 24, 12 males) were adolescents of Hungarian nationality recruited by a convenience sampling procedure. Age range was 15–22 years, while mean age was 18 years (SD: 2.3 years). The whole examined age range was subdivided into four subgroups (groups of 15–16, 17–18, 19–20 and 21–22 years old subjects). Six participants were included in each subgroup: 3 females and 3 males. Thus, subjects were evenly distributed over the age range. Mean height of the subjects was 173.04 cm (range: 160–198, SD: 10.57). Subjects’ weight averaged 63.83 kg (range: 47–92, SD: 11.92), while their body mass index (BMI) was between the normal limits (mean: 21.19, range: 17.68–27.01, SD: 2.6).

Subjects were interviewed on their health status by the authors of the study. Exclusion criteria for the participants were self-reported sleep problems or diagnoses of psychiatric, neurological or other medical disorders. Subjects were requested to not to drink alcohol containing beverages, to not to take drugs other than caffeine before noon and to not to take naps during the study.

The research protocol was approved by the Ethical Committee of the Pázmány Péter Catholic University Budapest. Adult participants or the parents of the underage participants signed informed consent for the participation in the study according to the Declaration of Helsinki.

### Procedures

Fluid intelligence was tested by using the Raven Progressive Matrices Test (RPMT), which is based on items assessing the abilities in the field of non-verbal reasoning (Raven et al., 1976). Scores of the RPMT were shown to be among the most reliable measures of the general factor of mental abilities (Gray and Thompson, 2004). Raw RPMT scores were transformed to IQ by using the Hungarian standards (Raven et al., 2004). As a consequence the term IQ reflects fluid instead of crystallized intelligence throughout our paper. Subjects’ sleep was recorded at their homes by using ambulatory home polysomnography. Sleep recordings on two consecutive weekend nights were performed according to the subjects’ sleeping habits. We used a portable SD LTM 32BS Headbox together with a BRAIN QUICK System PLUS software (Micromed, Italy) for polysomnographic data recording. We recorded EEG according to the 10–20 system (Jasper, 1958) at 21 recording sites (Fp1, Fp2, Fpz, F3, F4, F7, F8, Fz, C3, C4, Cz, P3, P4, Pz, T3, T4, T5, T6, O1, O2, Oz) referred to the mathematically linked mastoids. Bipolar EOG, ECG and submental as well as tibialis EMG were also recorded. Electroencephalogram and polygraphic data were high-pass filtered at 0.15 Hz and low-pass filtered at 250 Hz (both 40 dB/decade). Data were collected with an analog to digital conversion rate of 4096 Hz/channel (synchronous, 22 bit). A further 40 dB/decade anti-aliasing digital filter was applied by digital signal processing (firmware) which low pass filtered the data at 463.3 Hz before the decimation by a factor of 4, resulting in a sampling rate of 1024 Hz.

Sleep recordings of the second nights were visually scored according to standard criteria (Rechtschaffen and Kales, 1968) in 20 s epochs. The following definitions were used for sleep architecture evaluation: time in bed (as the time from lights out to final awakening), total sleep time (defined as the amount of sleep from sleep onset to final awakening), wake time after sleep onset (WASO, excluding wakefulness after the final awakening), sleep efficiency (calculated as the percent of sleep time without WASO divided by the time in bed), sleep latency (defined as the period between lights off and the first appearance of S2 sleep), non-rapid eye movement (NREM), Stage 1 (S1), S2, SWS (defined as the amount of time spent in Stages 3 and 4), rapid eye movement sleep (REM), REM latency (defined as the period between sleep onset and the first epoch scored as REM), number of sleep cycles (number of REM periods separated from each other by more than 15 min), average REM period duration (duration of REM sleep divided by the number of REM periods) and average sleep cycle duration in minutes (sleep time from the sleep onset to the end of the last REM period divided by the number of sleep cycles).

The 4 s epochs containing artifactual sleep EEG (movement, sweating or technical artifacts) were manually removed before further automatic sleep EEG analyses. One male subject was excluded from the below listed quantitative EEG analyses (but not from the above mentioned sleep architectural one) because of technical artifacts interfering with deliberate and reliable signal processing approaches.

The Individual Adjustment Method (IAM) of sleep spindle analysis (Bódizs et al., 2009) was used to unravel the potential peculiarities of NREM sleep (stages 2–4) EEG spindling. In short the principle of sleep spindle detection is the idea that individual spindles are those groups of waves which last at least 0.5 s and contribute to one or two of the major peaks in the 9–16 Hz average amplitude spectra of NREM sleep EEG. Individual-specific spectral peaks were formalized by calculating the zero crossing points of their second order derivatives. The lower frequency peak corresponds to SSs while the higher frequency peak to FSs. As a result, features like mean density (spindles/min), duration (s) and amplitude (µV) of SSs and FSs can be determined in an individual- and derivation-specific manner. The dominant individual-specific frequency (Hz) of SSs and FSs is inherently derivation-independent in the IAM procedure. Based on the derivation-specific data on density, duration and amplitude we created averages for five regions: all derivations (region-independent), frontal derivations (Fp1, Fp2, Fpz, F3, F4, F7, F8, Fz), centro-parietal derivations (C3, C4, Cz, P3, P4, Pz), temporal derivations (T3, T4, T5, T6) and occipital derivations (O1, O2, Oz). Region-specific averages were used for descriptive purposes while the region-independent average values were starting points of inferential statistics.

Additional analyses were based on Fast Fourier Transform-based measurement of binwise spectral power in the 8–16 Hz range of all-night average NREM sleep (stages 2–4) EEG covering alpha and sigma waves. In line with the relevant guidelines, spectral power was log-transformed before the statistical analyses (Pivik et al., 1993; Jobert et al., 2013). This transformation is required in order to normalize the distribution of power values. Besides log-transformation, z-scores of the 8–16 Hz spectra were also analyzed. This latter transformation is justified by the findings supporting the striking trait-like reliability (De Gennaro et al., 2005) and the marked sensitivity (Bódizs et al., 2012) of this sleep EEG scores expressing discrete frequency points of the individual shapes of the sleep EEG spectra. Both log-transformed power (10th base) and z-transformed normalization (x-m/SD) were used in separate statistical models. Our aim was to compare the results based on the more sophisticated IAM of sleep spindle analysis with the relatively simple spectral analysis. While IAM is sensitive to sleep spindle features at the individual frequencies, spectral power mapping is able to provide evidence for the importance of sleep spindle activity occurring at specific frequencies.

### Statistics

Descriptive statistics on IQ, as well as on sleep architecture and regional sleep spindling are provided. As for inferential statistics, we followed a top-down approach by using consecutive tests progressing from global to gender-specific and local effects. The average (region-independent) sleep spindle variables (frequencies, densities, durations and amplitudes of individual specific SSs and FSs) were correlated with the output variables (age and IQ) by using the Pearson product-moment procedure. In case of the emergence of a significant region-independent correlation the next step was to analyze sexual dimorphism of the relationship (by comparing correlations for females and males using the Fisher r-to-z transformation), as well as to depict the potential region-specificities of the significant global effects by subjecting the derivation-specific sleep spindle vs. output variable correlations to the procedure of descriptive data analysis (Abt, 1987) adapted to quantified neurophysiology with mapping (Abt, 1990; Duffy et al., 1990). This procedure tests the global null hypothesis (“all individual null hypotheses in the respective region are true”) at level *α* = 0.05, against the alternative that at least one of the null hypotheses is wrong. According to Abt (1987) and Duffy et al. (1990) local, uncorrected significances at the level of *α* = 0.05 (descriptive significances) define the Rüger’s areas (Rüger, 1978). If N is the number of electrodes in the Rüger’s area, the investigator is required to choose a minimal number of unspecified null hypotheses (M), less than N, to be nominally rejected at a new, more conservative *α* level. Typically the value M/N is 1/2 or 1/3. The corresponding new *α* levels for these values are *α*/2 = 0.025 and *α*/3 = 0.017, respectively. We will use an M/N value of 1/2 and a corresponding new *α* of 0.025 in our analyses. If any M values (half of the correlation coefficients if M/N = 1/2) within the Rüger’s area individually reach the new *α* level of significance the overall null hypothesis is rejected for the Rüger’s area at the 0.05 level. This means that for at least one EEG derivation in the Rüger’s area the relationship is significant, allowing the investigator to make global confirmatory statement with controlled uncertainty. In order to obtain a better localization of regions with significant correlations between sleep spindling and IQ the correlations were represented by significance probability maps (Hassainia et al., 1994). Finally, we tested the age-independence of the relationship between sleep spindling and IQ by recalculating the significant spindle-IQ correlations with the effects of age partialled out.

Binwise NREM sleep EEG spectral data between 8 and 16 Hz was correlated with age and with IQ in females and males by using the same methodology as described above.

## Results

### Fluid intelligence

Raven Progressive Matrices Test-derived IQ-scores of the sample resulted in a group average of 104.12 (range: 91–126, SD: 10.82). Neither age (*r* = 0.30; *p* = 0.15), nor weight (*r* = 0.13; *p* = 0.51), height (*r* = 0.14; *p* = 0.50) nor BMI (*r* = 0.06; *p* = 0.77) correlated significantly with IQ. Males and females did not differ in their general mental abilities (*t* = 0.31; *p* = 0.75).

### Sleep architecture and sleep spindling

Details on sleep architecture of our sample are depicted in Table 1. In short subjects had a normal sleep structure with 4–8 sleep cycles, an average total sleep time of 8.23 h, a sleep efficiency of 94.84%, over 59% of S2, 12% of SWS and 25% of REM sleep (Table 1).

**Table 1:** Descriptive statistics of sleep architectural variables*.

	Mean	Min	Max	SD
Time in bed (min)	521.65	399.00	639.33	59.29
Total sleep time (min)	494.33	368.33	617.00	54.60
Sleep efficiency (%)	94.84	85.25	99.09	3.36
Wake time (min)	27.31	4.33	85.00	19.03
Relative wake time (%)	5.15	0.90	14.74	3.36
WASO (min)	19.50	1.00	81.66	19.02
Sleep latency (min)	10.72	2.00	38.00	10.09
NREM time (min)	365.86	302.00	447.00	38.33
Relative NREM time (%)	74.16	66.28	81.99	4.00
S1 time (min)	10.68	3.00	33.66	6.36
Relative S1 time (%)	2.16	0.62	6.28	1.23
S2 time (min)	294.34	208.33	386.00	49.70
Relative S2 time (%)	59.59	43.61	75.83	7.93
SWS time (min)	60.83	3.00	162.33	37.15
Relative SWS time (%)	12.40	0.56	33.98	7.70
REM time (min)	128.47	66.33	170.00	27.35
Relative REM time (%)	25.83	18.00	33.71	4.00
REM latency (min)	79.02	44.66	150.00	28.08
Number of sleep cycles	5.08	4.00	8.00	0.97
Average REM period time (min)	25.63	15.66	34.75	5.23
Average sleep cycle time (min)	99.35	74.83	133.91	15.32

*S1—Stage 1 sleep; S2—Stage 2 sleep; SWS—slow wave sleep; WASO—wake after sleep onset.

Slow spindle densities, durations and amplitudes prevail in the frontal regions. In contrast densities, durations and amplitudes of FSs peak in the centroparietal area (Table 2).

**Table 2:** Descriptive statistics on sleep spindling*.

	Mean	Min	Max	SD
SS frequency (Hz)	11.28	9.88	13.10	0.83
Average SS density (spindles×min^−1^)	6.83	4.45	9.29	1.46
Frontal SS density (spindles×min^−1^)	7.14	5.17	9.18	1.14
Centroparietal SS density (spindles×min^−1^)	6.68	4.06	9.35	1.56
Temporal SS density (spindles×min^−1^)	6.73	4.00	9.60	1.70
Occipital SS density (spindles×min^−1^)	6.46	2.85	9.58	2.03
Average SS duration (s)	1.41	0.86	2.58	0.54
Frontal SS duration (s)	1.48	0.89	2.63	0.53
Centroparietal SS duration (s)	1.39	0.85	2.56	0.54
Temporal SS duration (s)	1.38	0.83	2.53	0.54
Occipital SS duration (s)	1.33	0.76	2.55	0.55
Average SS amplitude (µV)	3.48	1.30	6.91	1.65
Frontal SS amplitude (µV)	4.42	1.50	9.86	2.25
Centroparietal SS amplitude (µV)	3.64	1.39	7.21	1.72
Temporal SS amplitude (µV)	2.26	0.84	4.24	1.02
Occipital SS amplitude (µV)	2.34	0.80	4.89	1.10
FS frequency (Hz)	13.29	12.55	14.24	0.47
Average FS density (spindles×min^−1^)	7.27	5.50	8.29	0.75
Frontal FS density (spindles×min^−1^)	6.59	5.07	7.73	0.75
Centroparietal FS density (spindles×min^−1^)	8.11	6.58	9.74	0.75
Temporal FS density (spindles×min^−1^)	7.20	5.23	8.57	0.92
Occipital FS density (spindles×min^−1^)	7.45	4.86	9.01	0.93
Average FS duration (s)	1.10	0.89	1.29	0.09
Frontal FS duration (s)	1.02	0.86	1.16	0.07
Centroparietal FS duration (s)	1.21	0.97	1.45	0.10
Temporal FS duration (s)	1.07	0.88	1.29	0.09
Occipital FS duration (s)	1.15	0.86	1.40	0.11
Average FS amplitude (µV)	5.10	3.45	7.08	1.04
Frontal FS amplitude (µV)	4.88	2.95	7.02	1.08
Centroparietal FS amplitude (µV)	7.16	4.64	10.38	1.60
Temporal FS amplitude (µV)	3.26	2.42	4.37	0.50
Occipital FS amplitude (µV)	4.01	2.08	5.74	1.10

*SS—slow spindle, FS—fast spindle.

### Sleep spindling and age

**Figure 1:**
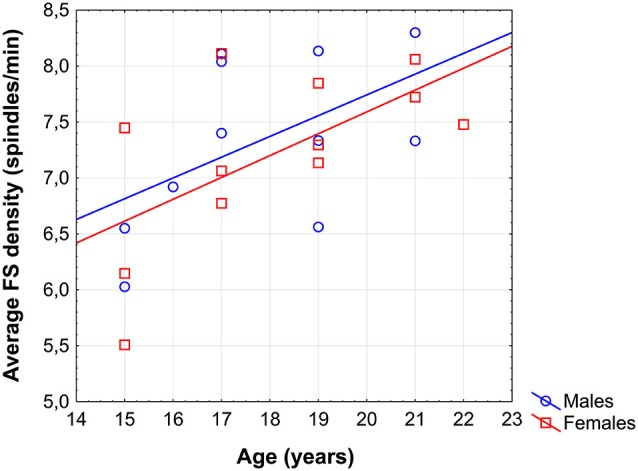
**Scatterplot revealing the age-dependent increase in sleep EEG fast spindle (FS) density of adolescents**.

Average FS density correlated positively with chronological age (*r* = 0.57; *p* = 0.005; Figure 1). No other sleep spindle measures were significantly related with the age of our subjects. There was no significant difference between the age vs. FS density correlations of females and males [*r* = 0.62 and *r* = 0.52, respectively; *p* = 0.76 (two-sided)].

**Figure 2:**
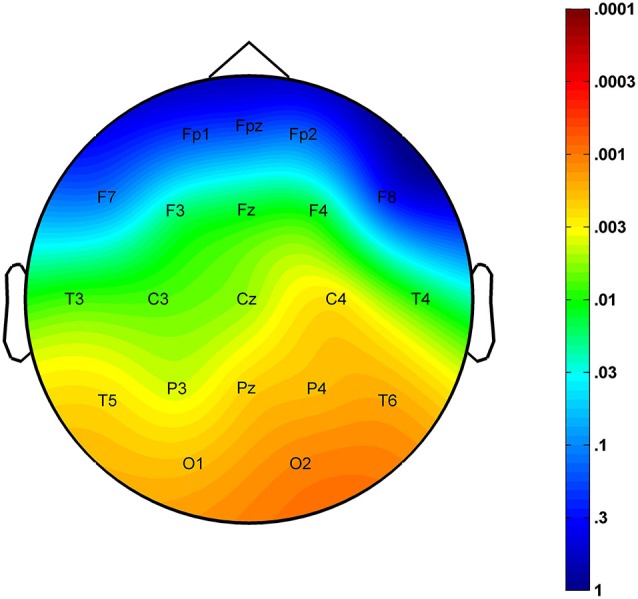
**Significance probability map for the region-specific correlations depicting the age-related increase in sleep EEG FS densities**. *P*-values are plotted on inverted logarithmic scale.

The region-specific analysis revealed a significant age-related increase in FS density measured at 16 of 21 derivations (F3, F4, Fz, C3, C4, Cz, T3, T4, T5, T6, P3, P4, Pz, O1, O2, Oz) defining a significant Rüger’s area (16/16 *p* values < 0.025) consisting of frontal, centroparietal, temporal and occipital regions, but not of frontopolar-orbitofrontal (Fp1, Fp2, Fpz, F7, F8) ones (Figure 2).

### Sleep spindling and IQ

Intelligence quotient was shown to be significantly and positively related to average FS density (*r* = 0.43; *p* = 0.04) and amplitude (*r* = 0.41; *p* = 0.049). While females were characterized by significant FS density vs. IQ, as well as FS amplitude vs. IQ correlations [*r* = 0.80 (*p* = 0.002) and *r* = 0.67 (*p* = 0.012)], respectively, males were not [*r* = 0.00 (*p* = 0.99) for both measures]. Differences between the correlation coefficients depicting the linear relationship between FS density vs. IQ of females and males was significant (*p* = 0.017, one-sided). However, the female-male difference in FS amplitude vs. IQ correlation proved to be a tendency only (*p* = 0.055, one-sided). One-sided statistics were used because of our explicit hypothesis on female predominance in the spindle vs. IQ correlations.

**Figure 3:**
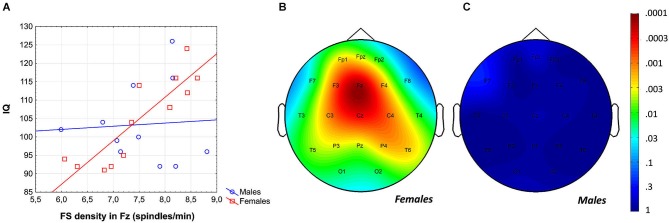
**Gender-specific sleep EEG FS density vs. IQ relationship in adolescents. (A)** Scatterplot representing the frontal midline FS density vs. IQ relationship. **(B)** Significance probability map of the FS density vs. IQ correlations in females.** (C)** Significance probability map of the FS density vs. IQ correlations in males. *P*-values are plotted on inverted logarithmic scale.

The region-specific analysis of the FS density vs. IQ correlation of females revealed significant correlations in 21 out of 21 derivations, 19 of which were significant at the level of 0.025 (Figure 3). Thus, findings fulfill the criteria for rejecting the global null hypothesis. Maximal significances were revealed over the frontal midline region (*r* = 0.90; *p* = 0.0001 at derivation Fz).

**Figure 4:**
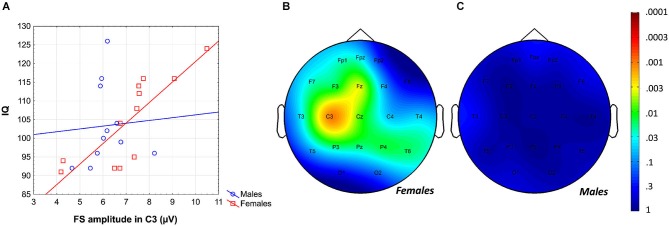
**Gender-specific sleep EEG FS amplitude vs. IQ relationship in adolescents**. **(A)** Scatterplot representing the frontal midline FS amplitude vs. IQ relationship. **(B)** Significance probability map of the FS amplitude vs. IQ correlations in females. **(C)** Significance probability map of the FS amplitude vs. IQ correlations in males. *P*-values are plotted on inverted logarithmic scale.

Likewise, the region-specific analysis of the FS amplitude vs. IQ correlation of females revealed significant correlations in 12 out of 21 derivations (Fp1, Fpz, F3, F7, Fz, C3, Cz, P3, P4, Pz, T3, T6), 8 of which were significant at the level of 0.025 (Figure 4). Again, based on these findings the global null hypothesis can be rejected. Maximal significances were revealed over the left central region (*r* = 0.82; *p* = 0.001 at derivation C3).

### Age-corrected relationships between sleep spindling and IQ

**Figure 5:**
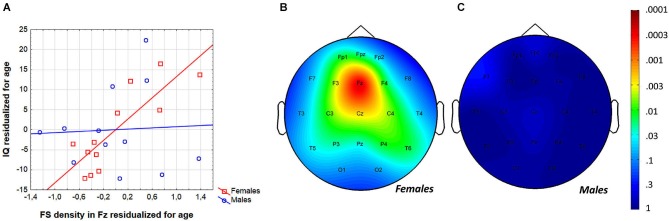
**Age-independence of the sleep EEG FS density vs. IQ relationship in females. (A)** Scatterplot representing the partial correlations between FS density and IQ (both were residualized for age). **(B)** Significance probability map of the FS density vs. IQ partial correlations (effects of age partialled out) in females. **(C)** Significance probability map of the FS density vs. IQ partial correlations (effects of age partialled out) in males. *P*-values are plotted on inverted logarithmic scale.

In order to test whether individual levels of fast sleep spindling age-independently predict general mental ability in adolescent females, partial correlations were calculated and entered in the procedure of descriptive data analysis and significance probability mapping (Figure 5). We found 13 significant correlations (out of 21) between FS density and IQ with the effects of age partialled out. The Rüger’s area consisted of a wide region including frontopolar-prefrontal, central, parietal and posterior temporal locations (Fp1, Fpz, F3, F4, Fz, C3, C4, Cz, T5, T6, P3, P4, Pz) with *p* values less than 0.025 at 11 derivations. Thus, the area includes significant FS density vs. IQ partial correlation (with the effects of age held constant) in adolescent females. Maximal correlation emerged at the frontal midline derivation Fz (*r* = 0.90; *p* = 0.0002).

The same analyses were run with FS amplitudes. Eight out of 21 partial correlations were significant in adolescent females, depicting a scattered parasagittal area (F7, Fz, C3, Cz, T6, P3, P4, Pz) with four *p* values being less than 0.025. Thus, the null hypothesis cannot be unambiguously rejected for this Rüger’s area.

### Are there any sleep spindle correlates of IQ in males?

**Figure 6:**
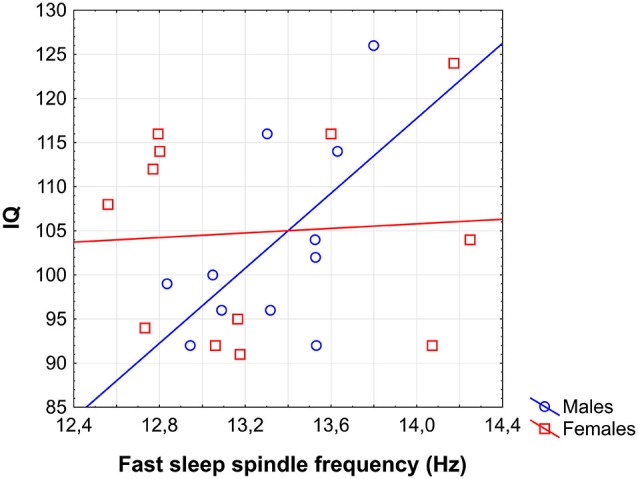
**Scatterplot representing the correlation between sleep EEG FS frequency and IQ in males**.

In previous analyses we progressed from global to sex-specific and local effects. This approach could hinder the recognition of some weaker, male-specific correlations between sleep spindles and IQ. In order to reveal any male-specific sleep spindle correlates of IQ in adolescents the correlations between all sleep spindle variables and IQ were checked for the male subgroup only. Analysis revealed a significant correlation of FS frequency with IQ in males (*r* = 0.60; *p* = 0.04; Figure 6). Partialling out the effects of age even slightly increased the strength of this relationship (*r* = 0.65; *p* = 0.04). No other correlation between sleep spindle measures and IQ in males proved to be significant.

### EEG sigma power

In females, neither log-transformed EEG powers nor z-scores revealed significant associations with IQ after the Rüger area correction, with or without control for the effects of age.

**Figure 7:**
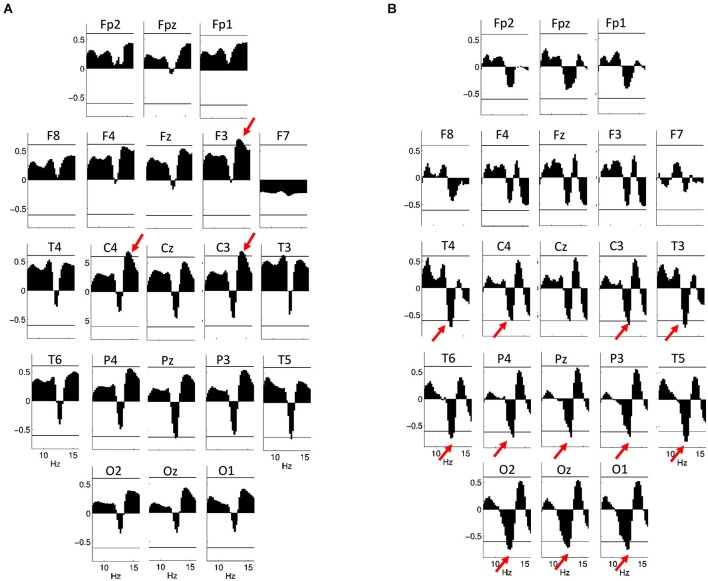
**Correlations between NREM sleep EEG spectral power of 8–16 Hz frequency and IQ in males**. Graphs are indicating region-specific correlations as revealed at different scalp locations. Horizontal lines denote critical values for *p* < 0.05. **(A)** Binwise spectral data were log-transformed (10th base) before implementing correlation analyses. Positive correlations of NREM sleep EEG 13.75–15 Hz spectral power at derivations F3, C3 and C4 with IQ (red arrows) are significant after controlling for multiple testing according to the procedure of descriptive data analysis. **(B)** Binwise spectral data were z-transformed before implementing correlation analyses. Negative correlations of NREM sleep EEG 12–13.25 Hz spectral power at derivations C3, C4, P3, P4, Pz, T3, T4, T5, T6, O1, O2 and Oz with IQ (red arrows) are significant after controlling for multiple testing according to the procedure of descriptive data analysis.

In males, however, a positive association between log-transformed EEG power on F3, C3 and C4 between 13.75 and 15 Hz (*r*_max_ = 0.70; *p* = 0.014 on F3 at 14 Hz) is significant after Rüger correction, while there is a tendency (with significant correlations not surviving Rüger correction) for a negative correlation between IQ and log-transformed power between 12.75 and 13 Hz on T5 and Pz (Figure 7A). Using EEG power z-scores, a significant negative correlation between IQ and power is present between 12 and 13.25 Hz on C3, C4, P3, P4, Pz, T3, T4, T5, T6, O1 and O2 (*r*_max_ = −0.78; *p* = 0.001 on T5 at 12.75 Hz; Figure 7B). Similar results were obtained if age-controlled correlations were used. In this case, no Rüger-significant effects are evident in females, while there is a significant negative correlation between IQ and power z-scores between 12 and 13.5 Hz (on C3, P3, P4, Pz, T3, T4, T5, T6, O1, O2, and Oz) in males. The positive correlation between IQ and log power is present between 13.75 and 15 Hz (on F3, C3, and C4) in males, but does not reach significance after correcting with the Rüger area method.

## Discussion

We performed a home polysomnographic study in order to unravel the developmental peculiarities of sleep spindling during adolescence as well as to test the predicted sexual dimorphism in the sleep spindle-IQ relationship during the period of the late maturation of the frontal lobes. Advantages of our study are the familiar, thus relatively non-disturbing sleeping environments and settings. Moreover, sleep was timed according to the preferred sleeping times of our subjects during two consecutive weekend nights. These circumstances are reflected in relatively long total sleep times (Table 1), at least when compared to laboratory based average values (Ohayon et al., 2004). Since longer sleep times lead to increases in S2 and REM sleep, the relative times spent in these two sleep stages were higher than usual while relative SWS times were lower. Given the fact that sleep spindles are most expressed in S2 sleep (De Gennaro and Ferrara, 2003) the above circumstances are not likely to mask the neurocognitive developmental aspects of sleep spindles.

Recent reports revealed the relationship between individual levels of sleep spindling and white matter integrity (Piantoni et al., 2013). Moreover, white matter continues to develop during late adolescence (Peters et al., 2012) resulting in continuously increasing integration and decreasing segregation of structural connectivity with age (Hagmann et al., 2010). We have shown that the prevalence (density) of centroparietally dominant FS of adolescents increases with age in both sexes, suggesting that “network refinement mediated by white matter maturation” (Hagmann et al., 2010) might be indexed by specific measures of sleep spindling (i.e., FS density). Thus, our current finding on the age-dependent increase in FS density in adolescents coheres with the above mentioned neuroimaging data (Hagmann et al., 2010; Peters et al., 2012; Piantoni et al., 2013) and strengthens/expands the reliability of the hypothesis suggesting that fundamental reorganization of cortical networks during adolescence is indexed by the changing patterns of synchronous, oscillatory activity (Uhlhaas et al., 2009; Konrad et al., 2013). Therefore, it is reasonable to assume that beside sleep EEG delta and theta activity indexing adolescent brain maturation (Feinberg and Campbell, 2013), sleep spindling is another neurophysiological marker with potential neurodevelopmental relevance. Given the widely accepted hypothesis on the thalamo-cortical origin of sleep EEG spindle oscillations (De Gennaro and Ferrara, 2003; Lüthi, 2014) the fundamental reorganization of the adolescent brain probably involves the developmental enhancement of the functionality of cortico-thalamic networks. As for the additional neurodevelopmental aspects of sleep spindling, it is worth noting, that the age-dependent increase of FS density during adolescence is the mirror image of the age vs. FS relationship of adult subjects, as the latter is characterized by a decline in spindling with increasing ages (Bódizs et al., 2009). Thus, the increasing FS density during adolescence suggests an inverted U-like relationship between age and fast sleep spindling during the human lifespan with maximal spindling emerging during the periods of maximal cognitive efficacy.

There are several previous studies investigating the relationship between cognitive abilities and sleep EEG spindling. Most of these studies are based on data from adult volunteers (Bódizs et al., 2005, 2008; Schabus et al., 2006, 2008; Lustenberger et al., 2012), some of them on investigations on preadolescent children (Geiger et al., 2011, 2012; Chatburn et al., 2013; Gruber et al., 2013), while none of them specifically addressed the period of late maturation of frontal lobes and related higher order cognitive functions. Here we aim to fill this gap by analyzing the period of adolescence and the transition from adolescence to adulthood from the perspective of sleep EEG spindle oscillation. Our present results on sleep spindle-IQ correlation and its predominantly frontal topography echoes previous findings (Bódizs et al., 2005; Fogel and Smith, 2011), further strengthens the primary role of frontal regions in intelligence (Gray and Thompson, 2004; Shaw et al., 2006), but also completes the picture with the issue of sex-specificity: FS density and amplitude was strongly and positively related with IQ in females only. Sleep spindles were shown to reflect the structural properties of white matter tracts (Piantoni et al., 2013). Thus, the female-specificity of the FS-IQ relationship reported here is reminiscent of earlier reports suggesting that anatomical measures of white matter structures are markers of cognitive ability in women, but not men (Gur et al., 1999; Haier et al., 2005). As white matter structures in fact serve efficient large-scale neural connectivity, the evidence indicating that EEG connectivity measures of the wakeful resting state are predictive of intelligence exclusively in women (Neubauer et al., 2002; Jausovec and Jausovec, 2005) might pertain in the same pattern of sexual dimorphism. In contrast with females, males were not characterized by a tight relationship of FS density or amplitude with IQ. Males, however, in contrast to females, were characterized by a positive FS frequency vs. IQ correlation. This was supported by spectral power data, which suggested a pattern of negative correlation between IQ and sigma power around 13 Hz as well as a positive correlation with higher sleep spindle frequencies around 14 Hz. Together, these results suggest that in adolescent males the tuning of sleep spindles to a higher, adult-like FS frequency is a more stable correlate of IQ than either amplitude or duration at the given individual frequency. While sleep spindle frequency has been shown to be a correlate of cognitive ability (Geiger et al., 2011; Bódizs et al., 2012), our results do not rule out the possibility that this correlation between IQ and spindle frequency is due to the effect of a maturation process which has already taken place in females of the same age.

Female sleep spindling frequency was shown to be influenced by the phase of the menstrual cycle (Ishizuka et al., 1994). As we did not control our subjects for the menstrual cycle phase effects this could hinder the depiction of the FS frequency-IQ relationship in females. Although, Tarokh et al. (2011) hypothesized that the increase of sleep spindle frequency during adolescence reflects the myelination of neural circuitry, there is no supporting evidence for this statement. However, we consider the above detailed sexually dimorphic correlations as further evidences for the fractionation of the general factor of intelligence into components (Conway and Kovács, 2013). Females, in contrast to males, rely on large-scale integration of neural circuitry during solving the complex non-verbal reasoning tasks of the RPMT. We hypothesize that this difference might emerge from different cognitive strategies of females and males. Indeed, there is evidence for certain sexual dimorphisms in cognitive strategies (Waller and Lin, 2012). Moreover, the report on the relationship between white matter structure and sleep spindling (Piantoni et al., 2013) together with our present finding on the relationship of individual level in FSs with IQ in females, but not in males serve as indirect evidences for the claim that women and men think with their white and gray matter, respectively (Zaidi, 2010).

Apart from the above mentioned difference between females and males other factors could contribute to the findings on the sexual dimorphism of the sleep spindle-IQ relationship of the present report. Among these factors the differences in the timing and the course of maturational processes (De Bellis et al., 2001) has to be mentioned.

There are several limitations of our study among which the relatively low number of subjects and the lack of longitudinal data must be mentioned. A higher number of subjects as well as a follow-up of our volunteers could provide a further refinement of our findings on the developmental aspects of sleep spindling and its relationship with general mental abilities in adolescents. Moreover, we did not monitor respiratory parameters and leg movements during sleep. Although sleep apnea and periodic leg movements during sleep are rare phenomena during adolescence we cannot completely rule out the possibility of the presence of these syndromes in some of our subjects.

To sum up our main empirical findings and conclusions we emphasize the following statements: (1) FS density is increasing during adolescent development; (2) FS density is an age-independent positive correlate of fluid intelligence in female adolescents. This latter effect is maximal over the frontal area; (3) FS frequency is a positive, age-independent index of fluid intelligence in male adolescents; (4) efficient network reorganization in the adolescent brain is indexed by specific, individually adjusted sleep spindle measures.

## Author contributions

Róbert Bódizs contributed to: the conception and design of the study, the visual scoring of sleep records, the quantitative analysis of sleep spindling, statistical analysis (descriptive, and sleep spindle-related inferential), interpretation of data for the work, designing and creating the figures of the manuscript, writing and critical review of the manuscript. Ferenc Gombos contributed to: the conception and design of the study, the quantitative analysis of sleep spindling and power spectra, interpretation of data for the work, designing and creating the figures of the manuscript, writing and critical review of the manuscript. Péter P. Ujma contributed to: statistical analysis of the spectral power measures, interpretation of data for the work, designing and creating the figures for the manuscript, writing and critical review of the manuscript. Ilona Kovács contributed to: the conception and design of the study, interpretation of data for the work, designing the figures of the manuscript, writing and critical review of the manuscript. All authors (Róbert Bódizs, Ferenc Gombos, Péter P. Ujma, Ilona Kovács) approved the final version of the manuscript to be published and agreed for all aspects of the work regarding accuracy and integrity.

## Conflict of interest statement

The authors declare that the research was conducted in the absence of any commercial or financial relationships that could be construed as a potential conflict of interest.

## Abbreviations

BMI: Body mass index
FS: Fast spindle
IAM: Individual Adjustment Method (of sleep spindle analysis)
RPMT: Raven Progressive Matrices Test
S2: Stage 2 sleep
SS: Slow spindle
SWS: slow wave sleep.
